# Rapid 3D imaging of the airway by MRI in patients with congenital heart disease: comparison of delayed volume interpolated breath hold examination (VIBE) technique to the turbo spin echo (TSE)

**DOI:** 10.1186/1532-429X-16-S1-P117

**Published:** 2014-01-16

**Authors:** Benjamin Goot, Sonali Patel, Brian Fonseca

**Affiliations:** 1 Pediatrics-Division of Cardiology, University of Colorado-Children's Hospital Colorado, Aurora, Colorado, USA

## Background

When imaging the airway by MRI, the traditional technique turbo spin echo (TSE) results in high quality 2D images of the airway. However, planning and acquisition times are often lengthy. An alternative technique for airway image acquisition, delayed volume interpolated breath hold examination or VIBE, is a 3D gradient echo technique that produces high spatial resolution imaging of the airway in one breath hold. The aim of this study was to compare airway measurements obtained by both TSE and delayed VIBE sequences in order to demonstrate that the quicker delayed VIBE produces a comparable evaluation of the airway to the standard technique.

## Methods

Patients with congenital heart disease, who underwent a cardiac MRI that included a delayed VIBE sequence from 5/2008 to the 9/2013, were included. Studies were performed on a 1.5-T Siemens Avanto (Siemens Medical Solutions, Erlagen, Germany). The TSE imaging was performed in the axial, sagittal and coronal planes per institutional protocol. The delayed VIBE (TR/TE 3.7/1.2 ms, flip angle 15o, voxel 1.5-1.8 × 0.8-1 × 1.0-1.2 mm) was acquired in the sagittal plane 5 minutes after gadolinium contrast administration. Two observers reviewed the studies (BG, BF) and airway measurements were made on both the delayed VIBE and TSE images in a blinded fashion to the other observer and other technique values. Intraclass correlations were calculated for agreement between both techniques, as well as between the two observers.

## Results

29 studies met inclusion criteria for analysis with a mean age of 8.8 years (2 months to 63 years) and mean weight of 30.2 kg (3.5-110). Of those 29 studies, 16 had both TSE and delayed VIBE sequences. The remaining 13 studies had no TSE imaging but the delayed VIBE measurements were included in the analysis for inter-observer variability. All delayed VIBE and TSE sequences were found to be of diagnostic quality. Mean acquisition time was significantly shorter for the delayed VIBE at 13.1 seconds (7.6-24.9) than TSE at 949.9 seconds (361-2318). Overall there was very good agreement between the delayed VIBE and TSE measurements (intraclass correlation coefficient (ICC) 0.94-0.78) with the exception of the distal right bronchus (ICC 0.67) The inter-observer agreement was also excellent for both TSE (ICC 0.94-0.78) and VIBE (ICC 0.96-0.89) (see Table 1).

**Table 1:** 

	Inter-technique ICC by Observer		Inter-observer ICC by Technique	
**Site**	**BG**	**BF**	**TSE**	**VIBE**

Proximal Trachea Major Axis	0.871	0.921	0.897	0.924

Proximal Trachea Minor Axis	0.899	0.919	0.917	0.921

Proximal Trachea Area	0.915	0.782	0.900	0.851

Distal Trachea Major Axis	0.782	0.900	0.938	0.903

Distal Trachea Minor Axis	0.810	0.878	0.886	0.932

Distal Trachea Area	0.783	0.882	0.914	0.948

Proximal Right Bronchus	0.936	0.801	0.956	0.955

Distal Right Bronchus	0.917	0.666	0.803	0.928

Proximal Left Bronchus	0.792	0.855	0.834	0.912

Distal Left Bronchus	0.890	0.795	0.779	0.894

## Conclusions

Delayed VIBE is a rapid and robust alternative to TSE imaging for assessment of the airway by MRI across a wide spectrum of patient age and size.

## Funding

This study was not funded and the author(s) declare no potential conflicts of interest with respect to the research, authorship, and/or publication of this article.

**Figure 1:**
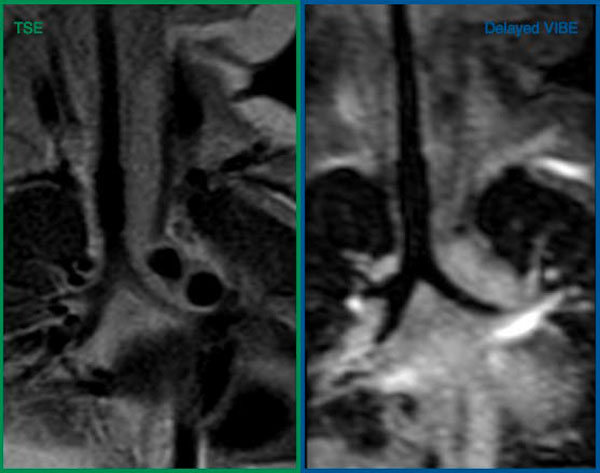
**Coronal images from a 1 year 11 month old child (7 kg) demonstrating the image quality obtained from both TSE and VIBE**.

